# The influence of social value orientation and group relations on fairness norms enforcement

**DOI:** 10.3389/fpsyg.2025.1684271

**Published:** 2025-10-29

**Authors:** Zhen Zhang, Chunhui Qi, Guoxiang Zhao

**Affiliations:** ^1^Faculty of Education, Henan University, Kaifeng, China; ^2^Faculty of Education, Henan Normal University, Xinxiang, China

**Keywords:** social value orientation, group relations, fairness norms enforcement, ultimatum game, group bias

## Abstract

Fairness norm enforcement represents a defining characteristic of human societies and is significantly influenced by group dynamics. However, the direction in which group relations influence the fairness norms enforcement remains controversial, and the underlying mechanism by which social value orientation modulates this effect has not yet been examined. A mixed experimental design with 2 (social value orientation: pro-socials vs. pro-selves) × 2 (group relationship: in-group vs. out-group) × 3 (proposal size: 5:5, 3:7, and 1:9) was employed to examine the impact of social value orientation and group relationship on the fairness norms enforcement during group resource allocation scenarios using a single anonymous ultimatum game. The results revealed that pro-socials were more likely to accept unfair distribution offers when interacting with in-group members compared to out-group members. However, no significant interaction effect between group relationship and proposal size on pro-selves’ acceptance rates was detected. Moreover, pro-selves responded significantly faster to the extremely unfair offers (1:9) when dealing with out-group members, whereas pro-socials exhibited shorter response times when interacting with in-group members. Responses to the other two distribution offers (3:7 and 5:5) were not significantly affected by either social value orientation or group relationship. Notably, for the 3:7 offers, pro-socials demonstrated higher in-group favoritism scores than pro-selves, while no such differences were observed for the 5:5 and 1:9 offers. These findings indicate that social value orientation and group relationship can jointly influence individuals’ normative responses to unfair distribution schemes.

## 1 Introduction

Fairness and justice are not only enduring moral ideals that guide human societies, but also serve as essential value standards in the process of modernizing national governance (Henrich et al., 2006; McAuliffe et al., 2017; Yang et al., 2024). Furthermore, they constitute a core element of contemporary socialist core values. When individuals encounter situations perceived as violations of fairness and justice, they often demonstrate a readiness to sacrifice personal gains to penalize the offenders. This behavioral tendency is referred to as fairness norm enforcement (Zhang et al., 2021, 2022). In recent years, researchers have employed well-structured economic game paradigms, such as the ultimatum game (UG) and third party punishment (TPP), to investigate how individuals perceive and react to unfair circumstances (Güth et al., 1982; Marshall and McAuliffe, 2022, 2024; McAuliffe et al., 2025). During the classical UG, two anonymous participants negotiate the division of a fixed monetary amount (e.g., 10 Yuan). The proposer suggests a distribution offer, and the responder may either accept or reject it. If accepted, both receive their respective shares; if rejected, neither gains anything. Empirical findings from UG studies consistently reveal that individuals generally exhibit a strong preference for equitable outcomes, reject unfair allocations, and are willing to uphold fairness norms at personal cost (McAuliffe et al., 2017; Zhang et al., 2021). To account for the rejection behavior observed in the Ultimatum Game task, numerous scholars across various disciplines have proposed theoretical perspectives including inequity aversion theory (Fehr and Schmidt, 1999), reciprocity theory (Falk and Fischbacher, 2006), emotional response theory (Van’t Wout et al., 2006), and dual-process theory (Bear and Rand, 2016). Moreover, evidence suggests that fairness norm enforcement has a significant genetic foundation (Wallace et al., 2007) and begins to emerge during early childhood, particularly between the ages of six and eight (McAuliffe et al., 2017). Additionally, this behavior is better understood through the lenses of cognition, emotion, and motivation, and is susceptible to influence by both personal factors and environmental factors (Güth and Kocher, 2014; Zhang et al., 2020; Qi and Zhang, 2020). It is important to recognize that while recent scholarship has proposed self-interested, spiteful, or competitive motivations as potential drivers of rejection behavior in UG tasks (Yamagishi et al., 2017a; Raihani and Bshary, 2019), the prevailing consensus among researchers still positions such behavior as a manifestation of fairness enforcement and a fundamental mechanism underlying the emergence and maintenance of cooperation (Henrich and Muthukrishna, 2021).

Human beings are inherently social creatures who reside within groups. Collaborative efforts and interpersonal interactions permeate all aspects of social life, and consequently, group dynamics significantly influence individuals’ social cognition and decision-making processes. In recent years, the impact of group relationship on the fairness norm enforcement has emerged as a prominent research topic, drawing attention from scholars across multiple disciplines and yielding substantial findings (Marshall and McAuliffe, 2022; Zhang et al., 2020). While researchers generally agree on the existence of group bias in the fairness norm enforcement, there is ongoing debate regarding the direction and scope of this effect. Most studies suggest that in-group identification fosters favoritism toward fellow in-group members, thereby weakening the fairness norm enforcement within their own group (McAuliffe and Dunham, 2016). Conversely, some scholars propose the occurrence of the black sheep effect (BSE), wherein in-group members who violate group norms face harsher sanctions, such as exclusion or ostracism (Wang et al., 2016), potentially reinforcing the fairness norm enforcement (Zhang et al., 2020). These conflicting research findings raise an important question: How do individuals perceive and respond to unfair behaviors exhibited by members of their own group? This dilemma reflects the interplay between in-group favoritism and the black sheep effect, as well as the tension between emotional attachment and moral judgment. However, much of the existing research has primarily focused on manipulating various group-related cues, with limited attention given to the potential influence of individual differences. Social value orientation (SVO), as a stable personality trait, refers to an individual’s preference in resource allocation between oneself and others in interdependent situations and is considered a key motivational factor in explaining cooperative and competitive behaviors (Murphy and Ackermann, 2014; Van Lange et al., 1997). Furthermore, some studies argue that prosocial behavior and parochialism are mutually reinforcing, with prosocial individuals demonstrating heightened levels of narrow altruism to support the group’s survival and development (Choi and Bowles, 2007; Rusch, 2014). Therefore, it is of critical importance to investigate whether and how social value orientations influence group biases during the fairness norms enforcement.

## 2 Literature review and research hypotheses

### 2.1 Group relationship and fairness norms enforcement

With the rapid development and increasing frequency of global communication, diplomacy, economic trade, and cultural exchange in contemporary society, the necessity and importance of cooperation and collaboration among individuals from different racial, social, or cultural groups have become increasingly evident. Group relationship refers to an individual’s subjective perception of belonging to a particular social group, along with the associated values, emotions, and behavioral tendencies tied to that affiliation (Yu et al., 2024; Zhang et al., 2022). When group identity is salient and socially recognized, individuals may exhibit group bias or parochialism; that is, they tend to demonstrate greater kindness, tolerance, and altruism toward in-group members, while displaying suspicion, indifference, or even hostility toward out-group members (Hewstone et al., 2002). In recent years, the influence of group relationship on the fair norm enforcement has emerged as a significant research topic, attracting interdisciplinary attention from fields such as management science (Su et al., 2023), evolutionary science (Delton and Krasnow, 2017), psychology (Marshall and McAuliffe, 2022), and neuroscience (Reimers et al., 2017), yielding a substantial body of research findings.

However, existing studies present certain contradictions and inconsistencies. The majority of empirical evidence suggests that individuals tend to be more lenient toward norm violations committed by in-group members, while a smaller body of research indicates that individuals may impose harsher punishments on such violations. To account for these conflicting findings, scholars have proposed three competing theoretical frameworks: social identity theory, the theory of bounded generalized reciprocity and normative focus theory. Social identity theory (SIT) posits that heightened group identification enhances individuals’ sense of belonging and loyalty to their in-group, leading to more favorable treatment of in-group members and more negative evaluations of out-group members (Tajfel, 1982). Consequently, group identity may undermine the impartial enforcement of norms, manifesting as in-group favoritism (Apps et al., 2018; Brüne et al., 2012; Guo et al., 2020; Kubota et al., 2013; Wang et al., 2017). The theory of bounded generalized reciprocity (BGR) posits that in-group favoritism stems from an evolutionarily adaptive decision heuristic, which drives individuals to establish and maintain a favorable reputation as cooperative partners (Yamagishi et al., 1999; Yamagishi and Kiyonari, 2000). By cultivating such a reputation, individuals increase their chances of securing indirect reciprocal benefits over time while simultaneously minimizing the risk of social exclusion within the group (Everett et al., 2015; Imada et al., 2024). As a result, in efforts to preserve or enhance collective group standing, individuals are more likely to display tolerance and understanding in response to norm violations perpetrated by in-group members. Both SIT and the BGR account for the emergence of in-group favoritism, yet they diverge fundamentally in their underlying mechanisms. SIT attributes in-group bias to social preferences arising from individuals’ psychological identification with a specific group, while BGR explains such behavior through strategic expectations grounded in patterns of direct and indirect reciprocity (Everett et al., 2015).

In contrast, normative focus theory (NFT) emphasizes that cooperative norms are central to the formation and maintenance of group cohesion. Deviant behaviors that violate these expectations are perceived as threats to group identity, prompting individuals to impose stricter sanctions on in-group members who violate norms (McAuliffe and Dunham, 2016), a phenomenon known as the black sheep effect (McLeish and Oxoby, 2011; Mendoza et al., 2014; Guo et al., 2022; Wu and Gao, 2018). Although the in-group favoritism and the black sheep effect represent opposing behavioral tendencies, both ultimately aim to strengthen group cohesion and ensure group stability and survival. Importantly, systematic reviews and meta-analytic studies consistently suggest that individuals are generally more tolerant of norm violations committed by in-group members, indicating that in-group favoritism is more prevalent than the black sheep effect (Balliet et al., 2014; Lazić et al., 2021; McAuliffe and Dunham, 2016; Zhang et al., 2020). Based on the above, we propose Hypothesis 1: Group identity influences individuals’ enforcement of fair norms, as evidenced by a greater willingness to accept unfair proposals from in-group members compared to out-group members.

### 2.2 Social value orientation as a moderator

Interdependent interest structures fundamentally shape asset allocation scenarios, where decision-makers’ differential prioritization of stakeholder interests systematically impacts cognitive appraisals and behavioral manifestations. Social Value Orientation (SVO), defined as an individual’s motivational framework governing resource distribution between self and others in interdependent contexts, bifurcates into two archetypes: pro-self orientation focused on personal gain maximization versus pro-social orientation emphasizing collective welfare (Murphy and Ackermann, 2014; Pletzer et al., 2018). This construct constitutes a pivotal determinant in cooperation-competition decision matrices (Zhang et al., 2014), exhibiting remarkable temporal stability (Murphy et al., 2011) and subconscious behavioral instantiation (Cornelissen et al., 2011). Experimental economics studies employing Ultimatum Game paradigms reveal pro-socials demonstrate heightened inequality aversion compared to pro-selves (Yang et al., 2018), concomitant with amplified negative affect and altered normative expectations during unfair allocation processing (Wang et al., 2017). Neuroscientific evidence further reveals that pro-socials exhibit greater negative feedback-related negativity and heightened activation in emotion-related brain regions (Haruno et al., 2014; Hu and Mai, 2021), and are more likely to reject unfair distribution proposals (Bieleke et al., 2017). Collectively, these findings suggest that pro-socials are more inclined to sacrifice personal gains in order to uphold fairness and normative standards. Based on the above, we propose Hypothesis 2: Pro-socials are more inclined than pro-selves to reject unfair proposals.

Furthermore, social value orientation delineates the manner in which individuals engage with other members of society and is closely associated with their interpersonal communication experiences. Yang et al. (2018) observed that social value orientation differentially influences the fairness norm enforcement across self-other decision-making scenarios. Specifically, fairness judgment standards and behavioral patterns remain relatively stable when pro-selves make decisions for themselves or for others, whereas pro-socials adopt more permissive fairness thresholds when acting on behalf of others. Crucially, prosocial dispositions and parochialism are symbiotically intertwined, as prosocial individuals exhibit heightened altruistic tendencies toward their in-group that are strategically oriented toward advancing collective survival and development (Choi and Bowles, 2007; Rusch, 2014). Empirical studies reveal that in intergroup Prisoner’s Dilemma paradigms, prosocial individuals disproportionately invest resources in the in-group allocation pool compared to self-oriented counterparts, thereby evidencing enhanced in-group favoritism (De Dreu, 2010; Salvati et al., 2022). Consequently, Hypothesis 3 is posited: Compared to egoists, pro-socials demonstrate heightened in-group favoritism, showing greater acceptance thresholds for inequitable distributions initiated by in-group constituents.

In summary, the present study employs the Triple-Dominance Scale (Murphy et al., 2011; Van Lange et al., 1997) to assess and categorize individuals as either pro-socials or pro-selves. Additionally, the Minimal Group Paradigm (MGP, Tajfel et al., 1971) is utilized to manipulate the group relationship between interacting parties. The central objective of this research is to examine how individuals with distinct social value orientations respond to inequitable resource distribution proposals made by in-group or out-group members.

## 3 Methods

### 3.1 Experimental design and participant

A mixed experimental design with a 2 (social value orientation: pro-socials vs. pro-selves) × 2 (group relationship: in-group vs. out-group) × 3 (proposal size: 5:5, 3:7, 1:9) factorial structure was employed. Social value orientation served as the between-subjects variable, whereas group relationship and proposal size were treated as within-subjects variables. The dependent variables included the acceptance rates, response times, and the in-group favoritism scores. Specifically, the in-group favoritism scores for different proposals were calculated by subtracting the acceptance rates of the corresponding proposals under the in-group condition from those under the out-group condition (Zhang et al., 2021).

A priori sample size estimation was conducted using G*Power 3.1 (Faul et al., 2007). Based on the assumption of a moderate effect size (*f* = 0.25), a significance level of α = 0.05, and the requirement for repeated measures analysis of variance between subjects, a minimum sample size of 28 participants was determined to achieve a statistical power of 95% (1 −β). A Triple-Dominance Scale (Murphy et al., 2011; Van Lange et al., 1997) was employed to conduct a preliminary assessment of the social value orientations among 78 undergraduate students. This measurement instrument comprises nine test items, each presenting three alternative options related to resource allocation between the self and anonymous others. These options represent three primary types of social value orientations: cooperative orientation, individualistic orientation, and competitive orientation. Participants were required to select their preferred resource allocation strategy from the three available options in each item. Subsequently, the number of selections corresponding to each of the three orientations was tallied. If a participant selected a particular orientation in more than six out of the nine items, that orientation was classified as their dominant social value orientation; otherwise, no definitive classification could be made. The results indicated that 38 participants exhibited a cooperative orientation, 19 demonstrated an individualistic orientation, 5 displayed a competitive orientation, and the social value orientation of 16 participants could not be definitively classified. Based on voluntary participation and availability, 24 cooperative individuals, 17 individualists, and 5 competitive-oriented participants were recruited for the subsequent experiment. Following the methodology of prior research (Murphy et al., 2011; Van Lange et al., 1997; Zhang et al., 2014), individualists and competitors were grouped together as pro-selves, while cooperator and altruist were grouped together as pro-socials. Thus, the final sample consisted of 24 pro-socials and 22 pro-selves. Among the participants, there were 6 male individuals, with a mean age of 20.34 ± 3.1 years. All subjects provided written informed consent prior to the experiment and received a predetermined amount of compensation upon completion of the study.

### 3.2 Manipulation and testing of group relationship

According to previous studies (Wang et al., 2017), the Minimal Group Paradigm was employed to manipulate the group relationship of the participants. Specifically, participants completed a computer-based task involving random group assignment. The task involved two cards displaying either red or blue spheres. Following a key-press by the participant, one card was selected, and participants were assigned to either the red group or the blue group based on the color of the spheres on the selected card. Although participants were informed that the card selection was random, the actual group assignment was predetermined by the experimental program to ensure a balanced distribution between the red and blue groups. To assess the effectiveness of the group manipulation, participants were asked to identify the group (red or blue) to which another participant belonged. Additionally, to evaluate participants’ perceived relationship with the in-group and out-group, two items from the Overlap of Self, In-group and Out-group scale (OSIO; Schubert and Otten, 2002) were used. Each item consisted of seven pairs of rings representing varying degrees of overlap between the self and either the in-group or the out-group. The degree of overlap was categorized into seven levels, with 1 indicating complete separation and 7 indicating maximum overlap. Participants were instructed to select the pair of rings that best represented their perceived relationship with the in-group and out-group.

### 3.3 Ultimatum game

According to previous studies (Yang et al., 2018), the ultimatum game utilized a single anonymous interaction format. Prior to the experiment, the research assistant informed participants that the research group had previously recruited 200 college students from various universities and assigned them to either the red group or the blue group through a standardized procedure. Participants were then instructed to assume the role of proposers and indicate their preferred allocation strategy in a one-time anonymous interaction with either an in-group or out-group member. The total allocation amount was fixed at 10 Yuan, and participants were required to choose among three allocation options: 5:5, 3:7, or 1:9 (where the number before the colon denotes the recipient’s share and the number after the colon denotes the proposer’s share). All decisions were stored in the computer system. During each round of the game, the program randomly selected one of the previously recorded allocation choices to execute the interaction. It is important to note that the presentation sequence of the allocation options was pseudo-randomly generated by the system.

### 3.4 Experimental procedure

Following the manipulation of group relationships, participants completed two sets of ultimatum game tasks: one involving intra-group interactions and the other involving inter-group interactions. Each set included five proposals for each of three allocation options presented in a sequential order to ensure balanced exposure across participants. Each trial commenced with a fixation cross displayed for a randomized duration between 400 and 800 ms, followed by the proposer’s allocation offer, which remained on screen for 1500 ms. Participants were instructed to make their decisions as quickly as possible within the allotted time, after carefully considering the offer. A key press of “1” signified acceptance, whereas a key press of “3” indicated rejection. The mapping of response keys was counterbalanced across participants to minimize response bias. In cases where no response was recorded within the time limit, the allocation offer was re-presented to allow for a second decision attempt. Upon completion of each trial, feedback was displayed on the screen, including color-coded avatars (red or blue) indicating group membership, the respective earnings for both parties in that trial, and the participant’s cumulative earnings. The formal experiment consisted of 30 trials. Prior to the main experiment, five practice trials were administered to familiarize participants with the experimental procedure. The entire experimental session lasted approximately 15 min. Each participant was provided with a baseline endowment of 5 Yuan and informed that additional rewards would be allocated based on their task performance. However, regardless of individual performance, all participants ultimately received an additional fixed reward of 5 Yuan.

## 4 Results

### 4.1 Manipulation check

First of all, all participants were capable of accurately identifying the group membership of the other party under both experimental conditions. Secondly, a mixed analysis of variance (ANOVA) was conducted on group overlap, with social value orientation and group relationship serving as independent variables. The results revealed that only the main effect of group relationship was statistically significant, *F*(1, 44) = 76.46, *p* < 0.001, η^2^ = 0.64. Specifically, the degree of overlap between the subject and the in-group (*M* = 4.02, SD = 0.20) was significantly higher than that between the subject and the out-group (*M* = 1.68, SD = 0.18). Therefore, the manipulation of the group relationship in the present study was effective.

### 4.2 Acceptance rates

A mixed analysis of variance was conducted with social value orientation, group relationship, and proposal size as independent variables to examine their effects on proposal acceptance rates. The results indicated a significant main effect of group relationship, *F*(1, 44) = 8.47, *p* < 0.01, η^2^ = 0.16. Specifically, the acceptance rate during in-group interactions (64.34 ± 2.22%) was significantly higher than that during out-group interactions (57.58 ± 2.24%). Additionally, the main effect of proposal size was also significant, *F*(2, 88) = 280.89, *p* < 0.001, η^2^ = 0.87. Post hoc comparisons revealed that the acceptance rates differed significantly among all pairs of the three proposal sizes, *p*s < 0.001, with acceptance rates decreasing sharply as the fairness of the distribution scheme declined. The interaction between group relationship and social value orientation was found to be significant, *F*(1, 44) = 10.89, *p* < 0.01, η^2^ = 0.20. Simple effect analysis indicated a significant group relationship effect for pro-socials, *F*(1, 44) = 20.16, *p* < 0.001. Specifically, the acceptance rate during in-group interactions (67.78 ± 3.07%) was significantly higher than during out-of-group interactions (53.33 ± 3.10%). In contrast, no significant difference was observed between in-group (60.91 ± 3.21%) and out-group (61.82 ± 3.24%) acceptance rates for pro-selves, *F*(1, 44) = 0.07, *p* > 0.05. The interaction between group relationship and proposal size was also significant, *F*(2, 88) = 3.75, *p* < 0.05, η^2^ = 0.08. Further analysis revealed a significant group relationship effect under the offer 3:7, *F*(1, 44) = 7.42, *p* < 0.01, with higher acceptance rates for in-group interactions (80.57 ± 4.37%) compared to out-group interactions (66.10 ± 5.62%). However, no significant group relationship effects were observed for the 1:9 and 5:5 offers, *F*s(1, 38) < 2.00, *p*s > 0.05.

**FIGURE 1:**
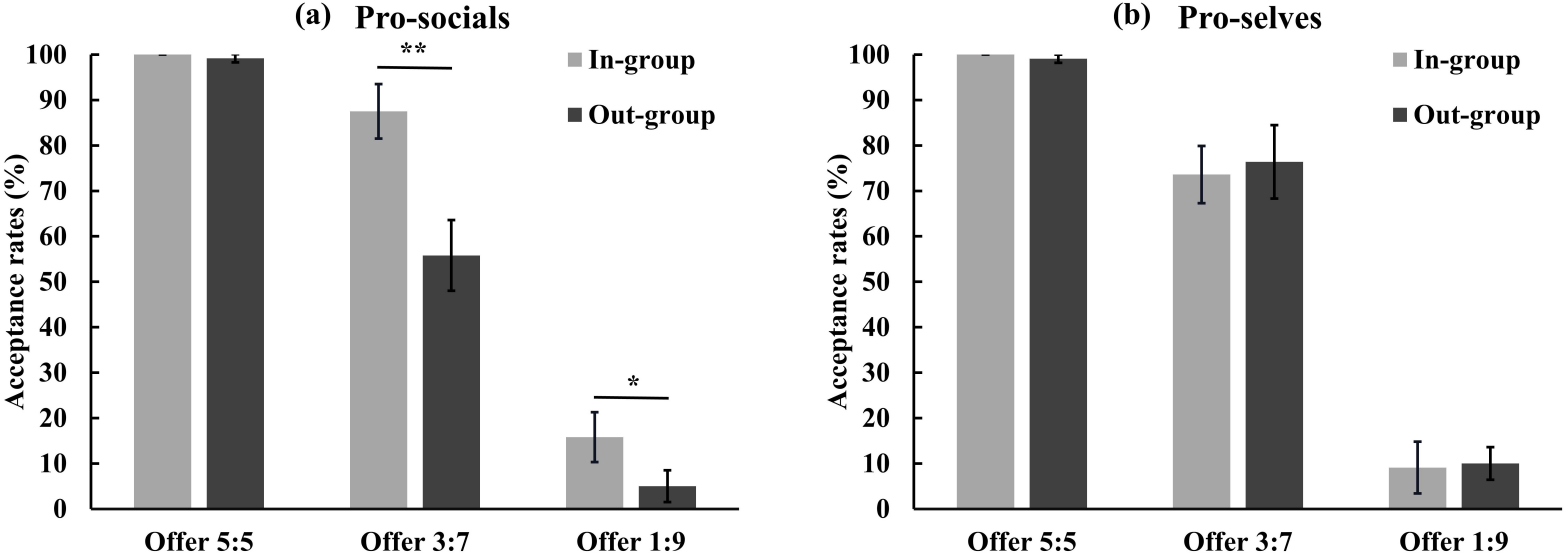
Averaged acceptance rates of different proposal as a function of group relationship for panel **(a)** pro-socials and **(b)** pro-selves. Error bars indicate SE. Asterisks indicate significant effects, *p < 0.05, **p < 0.01.

Notably, a significant three-way interaction among social value orientation, group relationship, and proposal size was observed, *F*(2, 88) = 5.90, *p* < 0.01, η^2^ = 0.12. Simple effect analysis revealed a significant interaction between group relationship and proposal size among pro-socials, *F*(3, 132) = 13.88, *p* < 0.001. Further analysis showed that group relationship significantly influenced the acceptance rates of the 1:9 and 3:7 offers, Fs(1, 44) > 4.50, ps < 0.05. Specifically, in-group interactions yielded higher acceptance rates (15.83 ± 5.49%, 87.50 ± 6.04%) than out-group interactions (5.00 ± 3.48%, 55.83 ± 7.77%) for these unfair offers. However, no significant effect of group relationship was found for the 5:5 offers, *F*(1, 44) = 0.96, *p* > 0.05. Moreover, no significant interaction between group relationship and proposal size was observed for pro-selves, *F*(3, 132) = 0.10, *p* > 0.05 (see Figure 1). All other main effects and interactions were not statistically significant.

### 4.3 Reaction times

**FIGURE 2:**
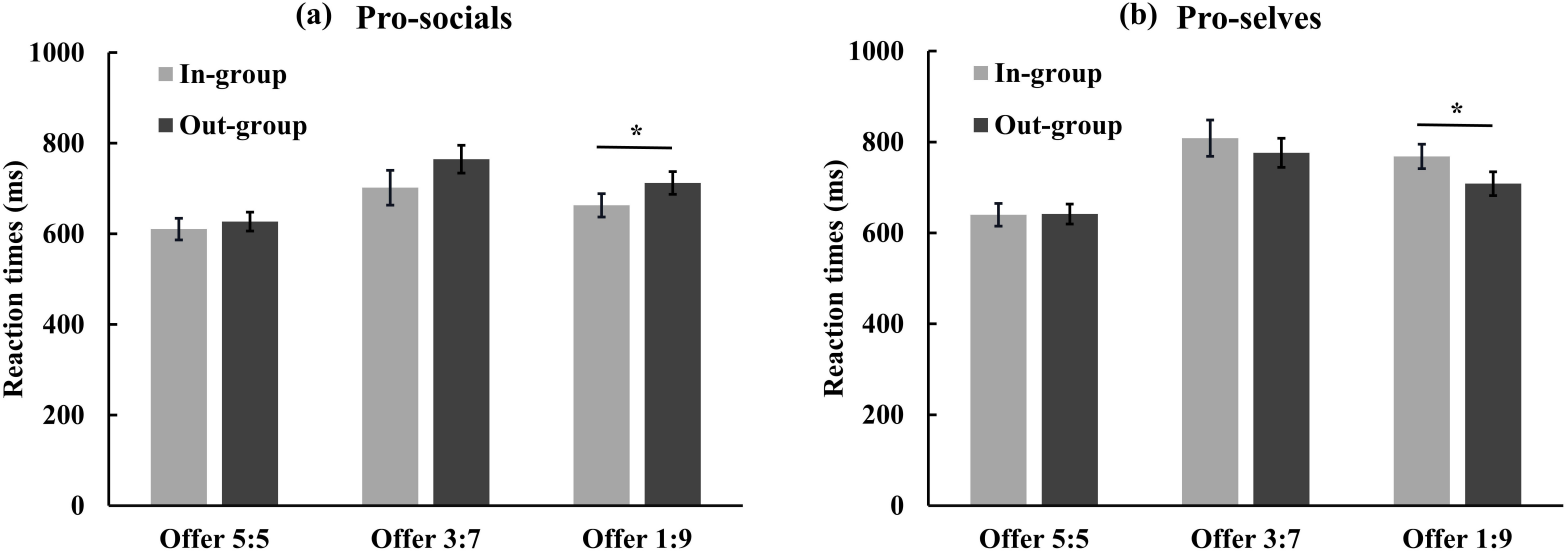
Averaged reaction times of different proposal as a function of group relationship for panel **(a)** pro-socials and **(b)** pro-selves. Error bars indicate SE. Asterisks indicate significant effects, *p < 0.05.

A mixed analysis of variance was conducted with social value orientation, group relationship, and proposal size as independent variables to examine their effects on reaction times. The results revealed a significant main effect of the proposal size, *F*(2, 88) = 32.02, *p* < 0.001, η^2^ = 0.42. Post hoc comparisons indicated significant differences in reaction times across all pairs of allocation schemes (ps < 0.05). Furthermore, the relationship exhibited an inverted U-shape pattern, with the shortest reaction times observed under the 5:5 offer (629.84 ± 14.60 ms), followed by the 1:9 offer (713.04 ± 16.18 ms), and the longest under the 3:7 offer (762.88 ± 21.52 ms). The interaction between social value orientation and group relationship was statistically significant, *F*(1, 44) = 6.58, *p* < 0.05, η^2^ = 0.13. Simple effects analysis demonstrated that pro-socials exhibited a significant group relationship effect, *F*(1, 44) = 4.76, *p* < 0.05. Specifically, reaction times were significantly shorter during in-group interactions (658.43 ± 24.25 ms) compared to out-group interactions (701.47 ± 21.05 ms). Conversely, no significant difference was observed for pro-selves during in-group (738.98 ± 25.32 ms) versus out-group interactions (708.81 ± 21.98 ms), *F*(1, 44) = 2.14, *p* > 0.05. A significant linear trend emerged in the three-way interaction among social value orientation, group relationship, and allocation scheme, F_*linear*_(1, 44) = 5.66, *p* < 0.05, η^2^ = 0.11. Additional simple effects analysis under the 1:9 offer indicated a significant interaction between social value orientation and group relationship, *F*(2, 44) = 5.00, *p* < 0.05. For the 1:9 offer, pro-selves’ reaction times for out-group recipients (708.45 ± 26.16 ms) were significantly shorter than for in-group recipients (768.46 ± 26.86 ms), *F*(1, 44) = 5.75, *p* < 0.05. Moreover, pro-socials displayed faster responses to the 1:9 offer when initiated by in-group members (662.92 ± 25.71 ms) versus out-group members (712.35 ± 25.05 ms), *F*(1, 44) = 4.26, *p* < 0.05. No significant interactions between social value orientation and group relationship were observed for the 3:7 or 5:5 offers, Fs(1, 44) < 1.91, ps > 0.05 (see Figure 2). All remaining main effects and interactions were non-significant.

### 4.4 In-group favoritism scores

**FIGURE 3:**
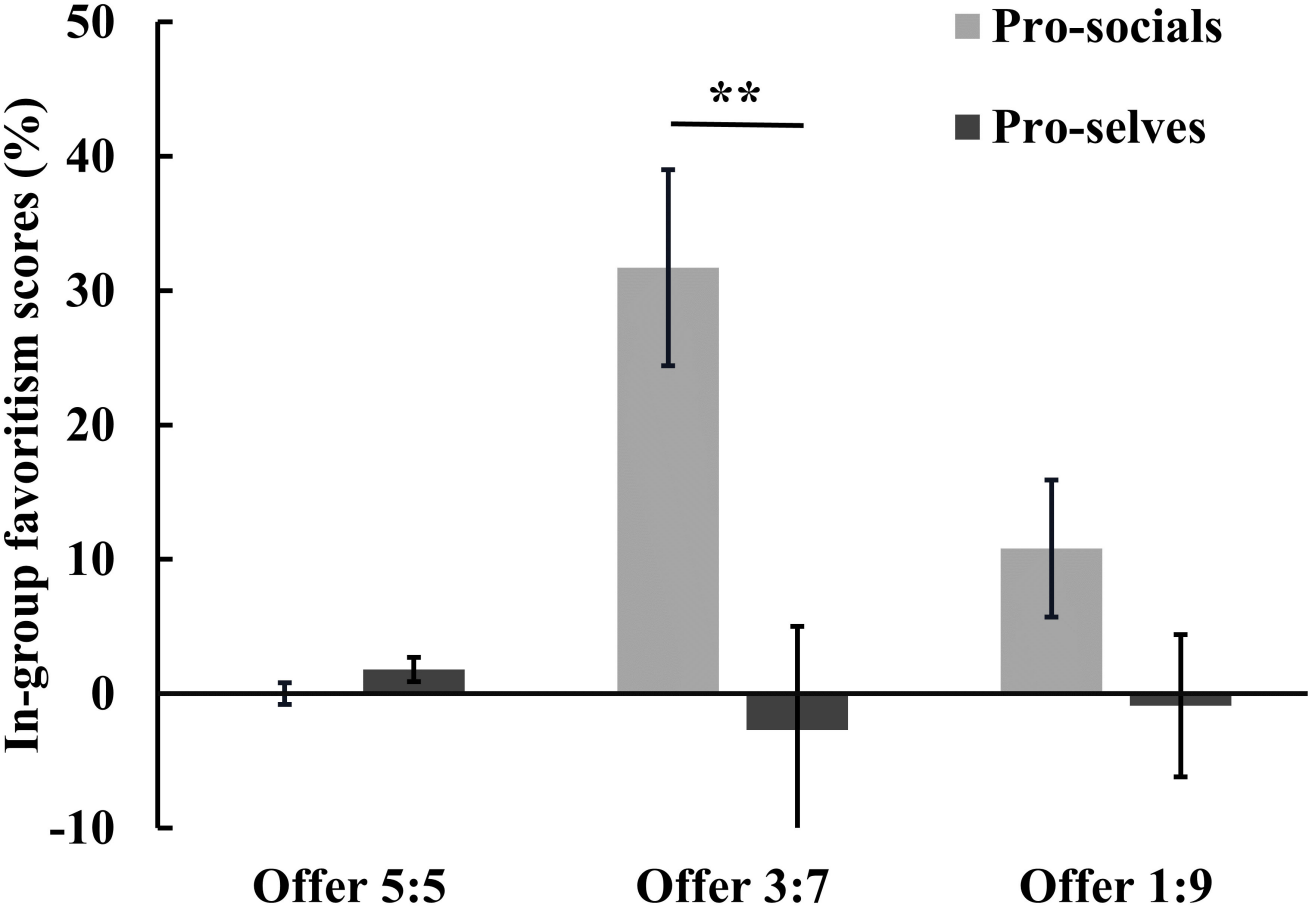
Averaged in-group favoritism score of different proposal for pro-socials and pro-selves. Error bars indicate SE. Asterisks indicate significant effects, **p < 0.01.

A mixed analysis of variance was conducted with social value orientation and proposal size as independent variables to examine the in-group favoritism scores. The results indicated a significant main effect of social value orientation, *F*(1, 44) = 9.75, *p* < 0.01, η^2^ = 0.18. Specifically, the in-group preference score of pro-socials (14.17 ± 3.27%) was significantly higher than that of pro-selves (−0.61 ± 3.42%). A significant main effect of the proposal size was also observed, *F*(2, 88) = 3.81, *p* < 0.05, η^2^ = 0.08. Post hoc comparisons revealed that the in-group favoritism scores for the 3:7 offer (14.47 ± 5.31%) was significantly higher than that for the 5:5 offer (0.91 ± 0.60%), *p* < 0.05. However, the in-group favoritism scores for the 1:9 offer (4.96 ± 3.69%) did not significantly differ from either of the other two offers, *p*s > 0.05. The interaction between social value orientation and proposal size was also significant, *F*(2, 88) = 6.59, *p* < 0.01, η^2^ = 0.13. A simple effects analysis was conducted based on social value orientation. The results showed that for the 3:7 offer, pro-socials exhibited significantly higher in-group favoritism scores (31.67 ± 7.34%) than pro-selves (2.73 ± 7.67%), *F*(1, 44) = 10.49, *p* < 0.01. However, no significant effect of social value orientation was found for the 1:9 and 5:5 offers, *F*s(1, 44) < 2.53, *p*s > 0.05 (see Figure 3). Another simple effects analysis was conducted based on the proposal size. The results revealed a significant effect of proposal size among pro-socials, *F*(1, 44) = 9.70, *p* < 0.001. Specifically, the in-group favoritism scores for the 3:7 offer (31.67 ± 7.34%) was significantly higher than those for the 1:9 (10.83 ± 5.11%) and 5:5 (0.00 ± 0.83%) offers; however, the difference between the latter two was not statistically significant, *p* > 0.05. In contrast, no significant effect of proposal size was observed among pro-selves, *F*(1, 44) = 0.24, *p* > 0.05.

## 5 Discussion

Current research has demonstrated that the acceptance rates of proposal size declines rapidly as the fairness of these offers diminishes, thereby reaffirming the central role of fairness in such contexts. This finding aligns with previous studies (McAuliffe et al., 2017; Yang et al., 2018), indicating that fairness and justice constitute fundamental principles guiding human social interactions. Individuals exhibit a strong preference for equitable outcomes and consider the interdependent interests and equality between parties involved. When confronted with situations perceived as unfair or in violation of established fairness norms, individuals are often willing to sacrifice their own potential gains to enforce punitive measures (Zhang et al., 2022). Furthermore, in the context of inter-group interactions, a significant in-group favoritism is observed in the acceptance of distribution offers. Specifically, individuals tend to be more accepting of allocation proposals made by in-group members, particularly when these proposals are ambiguous or moderately unfair. This observation is consistent with a substantial body of prior research (Apps et al., 2018; Brüne et al., 2012; Guo et al., 2022; Kubota et al., 2013; Wang et al., 2017), supporting the theoretical framework of SIT (Tajfel, 1982) and BGR (Yamagishi et al., 1999). When group distinctions and intergroup relationships become more salient, individuals not only develop a stronger sense of collective identity but also exhibit heightened sensitivity to the reputational implications of their behavioral choices. Consequently, they are more inclined to tolerate or rationalize moderate deviations from fairness, leading to a greater willingness to accept moderately unfair allocation schemes proposed by in-group members.

More importantly, social value orientation can moderate the phenomenon of in-group favoritism in the enforcement of fairness norms. On the one hand, there is a significant interaction between social value orientation and group relations. Further analysis indicates that when individuals enforce fairness norms, the influence of group relations varies depending on the individual’s social value orientation. Specifically, prosocial individuals exhibit a significantly higher acceptance rate of distribution offers proposed by in-group members compared to those proposed by out-group members. On the other hand, a simple effect analysis of the interaction among the three variables reveals that this difference primarily arises because prosocial individuals are more likely to accept unfair distribution schemes when they are proposed by in-group members. In contrast, self-interested individuals do not adjust their fairness norms enforcement based on the group affiliation of their interaction partners. Instead, prosocial individuals apply differentiated norms according to the group relationships involved and tend to impose less punishment on in-group members in unfair situations. This finding aligns with research in the field of interpersonal (Yang et al., 2018; Qi and Zhang, 2020) and inter-group interaction (De Dreu, 2010; Salvati et al., 2022). The result may be attributed to the fact that prosocial individuals possess superior theory-of-mind abilities and emotional regulation skills, which enable them to demonstrate greater situational sensitivity and adapt their enforcement of fairness norms in response to changes in group dynamics. Furthermore, this result partially supports the parochial altruism theory (Choi and Bowles, 2007; Rusch, 2014), suggesting that prosocial individuals exhibit stronger in-group favoritism. Such behavior may contribute to the survival, cohesion, and reproduction of the group.

Early work argued that faster reaction times reflected intuitive processes, while slower response times reflected deliberation (Rand et al., 2012). However, later studies and replications found that this link is not consistent across contexts (Bouwmeester et al., 2017), with a comprehensive meta-analysis by Kvarven et al. (2020b) failing to detect a statistically significant relationship between the two variables. While some empirical and meta-analytic studies propose that intuitive processes may be associated with tendencies toward pure cooperation (Rand, 2016) or self-preservation instincts (Capraro, 2024), these interpretations are subject to ongoing debate due to mixed evidence and methodological limitations (Kvarven et al., 2020a). Nevertheless, a robust body of scholarly work converges on the conclusion that longer reaction times reflect increased cognitive effort in resolving conflicting response options. Consistent with previous studies (Wang et al., 2017), the response time associated with the distribution offer exhibits an inverted U-shaped pattern, suggesting that moderately unfair offers induce greater ambiguity, heightened cognitive conflict, and require increased cognitive processing time. Current research indicates that pro-selves’ reaction times in response to distribution offers are not significantly influenced by group relationship. However, prosocial individuals demonstrate significantly longer reaction times when interacting with out-group members compared to in-group members. This implies that prosocial individuals tend to prioritize group interests when interacting with in-group members, resulting in fewer cognitive conflicts and shorter reaction times (Zhang et al., 2021).

More notably, the three-way interaction analysis revealed distinct response patterns between prosocial and pro-self individuals in response to highly unfair distribution schemes in inter-group contexts. Specifically, pro-selves exhibited significantly longer response times when presented with highly unfair offers from in-group members compared to out-group members. In contrast, pro-socials responded significantly faster to highly unfair offers from in-group members than from out-group members. These findings may be attributed to the differing strategies employed by prosocial and pro-self individuals when confronted with highly unfair proposals in inter-group settings (Yamagishi et al., 2017b). Specifically, prosocial individuals, who prioritize group harmony, demonstrate greater proficiency in rationalizing and regulating their emotional responses upon encountering unfair offers from in-group members, thereby experiencing reduced cognitive dissonance. Conversely, pro-self individuals, who prioritize personal gain, perceive highly unfair offers from in-group members as a significant threat to their self-interest, leading to heightened negative emotions and prolonged decision-making processes.

In addition, it is worth noting that current research has identified several noteworthy phenomena. Specifically, behavioral responses and ingroup favoritism scores were particularly pronounced at the 3:7 offer, whereas response times yielded significant results at the 1:9 offer. Two potential explanations may account for these findings. On the one hand, from the perspective of resource allocation fairness, the 3:7 offer represents a moderately unfair and relatively ambiguous condition, which tends to elicit greater individual variability in responses. Prior studies have consistently demonstrated that ingroup favoritism is more likely to emerge under such ambiguous and moderately unfair conditions (Gong et al., 2017; Mendoza et al., 2014). In this context, acceptance rate metrics are sufficiently sensitive to reflect the underlying motivations of individuals with varying social value orientations. In contrast, the 1:9 allocation is widely perceived as grossly unfair, thereby reducing variability in acceptance decisions. As a result, acceptance rates lose discriminative power, and reaction time emerges as a more sensitive measure, capturing subtle cognitive dissonance or residual in-group biases that persist despite the overt inequity of the proposal. On the other hand, although social value orientation (SVO) is considered a relatively stable personality trait, individuals with differing SVO profiles may still exhibit variability in interpersonal interactions. Research has shown that the relationship between reaction time and behavior is weaker among individuals with weak pro-social or weak pro-self orientations compared to those with consistent pro-social or consistent pro-self orientations, despite equivalent levels of behavioral variance (Yamagishi et al., 2017b). Empirical studies and meta-analyses have further demonstrated that shorter reaction times are associated with stronger cooperative behavior among pro-social individuals relative to pro-self individuals (Andrighetto et al., 2020; Sun et al., 2023).

Finally, pro-socials exhibit higher in-group favoritism scores than pro-selves, indicating a greater prioritization of collective interests and a stronger in-group orientation. Furthermore, this in-group favoritism predominantly emerges under the moderately unfair distribution offer of 3:7, which is consistent with prior research (Wang et al., 2017). This suggests that the 3:7 offer is marked by high ambiguity and uncertainty, thereby functioning as a distribution model with greater response variability and a greater likelihood of revealing in-group bias. Additionally, social value orientation and group relation interact to shape in-group favoritism scores. From the perspective of social value orientation, the analysis further demonstrates that prosocial individuals exhibit significantly higher in-group favoritism scores for the 3:7 offer compared to egoistic individuals, reinforcing the idea that ambiguous and moderately unfair scenarios elicit greater decision variability. Under such conditions, the heightened capacity for cooperation, empathy, and emotional regulation among prosocial individuals becomes more evident (Karagonlar and Kuhlman, 2013).

## 6 Implications and limitations

The present study represents the first empirical examination of the moderating role of social value orientation in the manifestation of group bias during the fairness norms enforcement. The findings demonstrate that individuals with pro-social orientations exhibit a stronger in-group favoritism compared to those with pro-self orientations. These results hold both theoretical and practical implications. Theoretically, they contribute to a clearer understanding of the directionality of group bias in norm enforcement contexts, revealing that individuals are more likely to tolerate norm violations when committed by in-group members, especially moderately vague and unfair proposals. Practically, the findings align with the central tenets of narrow altruism theory, suggesting a positive association between prosocial tendencies and in-group favoritism, which may enhance group cohesion, survival, and reproductive success. Furthermore, these insights facilitate a deeper comprehension of the underlying mechanisms and boundary conditions of in-group preference in the context of fairness norm enforcement, offering valuable perspectives for both academic research and real-world applications.

Similar to other studies, the present research also has certain limitations. First, the study employed a Minimal Group Paradigm to manipulate group relationship, which may deviate from real-world social interactions and thus lacks ecological validity (Lane, 2016). Future research should further validate the findings within more realistic group contexts. Second, while the study examined the influence of social value orientation on group bias under the fair norm enforcement, the underlying mechanisms remain unexplored (Zhang et al., 2022). Future studies may consider integrating cognitive or emotional factors as potential mediating variables. Third, the current research only investigated the second-party punishment scenario, in which participants acted as victims, and did not examine the third-party punishment scenario, in which participants serve as bystanders (Marshall and McAuliffe, 2022). Future research could extend the investigation to such scenarios to test the generalizability of the findings.

## 7 Conclusion

Social value orientation plays a pivotal role in determining the extent to which individuals exhibit in-group favoritism during the enforcement of fairness norms. Pro-social individuals are significantly more inclined to display a pronounced in-group bias when confronted with moderately unfair distribution schemes, whereas such bias does not manifest under conditions of fair offers. In contrast, pro-self individuals do not exhibit a notable in-group favoritism across all types of distribution schemes. Importantly, the difference in in-group favoritism scores between pro-social and pro-self individuals becomes apparent solely under moderately unfair conditions.

## Data Availability

The raw data supporting the conclusions of this article will be made available by the authors, without undue reservation.
